# The Soft and High Actuation Response of Graphene Oxide/Gelatin Soft Gel

**DOI:** 10.3390/ma14247553

**Published:** 2021-12-09

**Authors:** Supanit Chungyampin, Sumonman Niamlang

**Affiliations:** Department of Materials and Metallurgical Engineering, Faculty of Engineering, Rajamangala University of Technology Thanyaburi (RMUTT) Klong 6, Thanyaburi, Pathum Thani 12120, Thailand; supanit_c@mail.rmutt.ac.th

**Keywords:** soft hydrogel, actuators, electromechanical properties, dielectrophoresis force

## Abstract

The high actuation response of soft gel from a graphene oxide/gelatin composite was prepared as an alternative material in soft robotics applications. Graphene oxide (GO) was selected as the electroresponsive (ER) particle. GO was synthesized by modifying Hummer’s method at various ratios of graphite (GP) to potassium permanganate (KMnO_4_). To study the effect of ER particles on electromechanical properties, GO was blended with gelatin hydrogel (GEL) at various concentrations. The electrical properties of the ER particles (GO and GP) and matrix (GEL) were measured. The capacitance (C), resistance (R), and dielectric constant of the GO/GEL composite were lower than those of the GO particles but higher than those of the GEL and GP/GEL composite at the given number of particles. The effects of external electric field strength and the distance between electrodes on the degree of bending and the dielectrophoresis force (F_d_) were investigated. When the external electric field was applied, the composite bent toward electrode, because the electric field polarized the functional group of polymer molecules. Under applied 400 V/mm, the GO/GEL composite (5% *w*/*w*) showed the highest deflection angle (θ = 82.88°) and dielectrophoresis force (7.36 N). From the results, we conclude that the GO/GEL composite can be an alternative candidate material for electromechanical actuator applications.

## 1. Introduction

Recently, electromechanical actuators devices are used in a variety of applications, including microrobotic devices (exploration and repair of the human body), microscopic machines, spacecraft, robotics, and intelligent artificial muscles. For more concrete electromechanical actuator devices, shorter range goals, low cost, fast response, and reduced size and mass are required parameters [1].

Electroactive materials can be useful in the application of intelligent artificial muscles, living-thing-like actuators, and robotics. Recently, converting electrical energy into mechanical energy has been of interest. One kind of electroactive material is the electroactive polymers (EAPs), which have unique properties including light weight, flexibility, and high energy density [2].

EAPs use electrically stimulated polymers to change their size and/or shape and can mimic the behavior of muscles. EAPs can be divided into two main groups: electronic electroactive polymers (EEPs) and ionic electroactive polymers (IEAs). EEPs convert electricity into mechanical responses. The dimensional changes in the EEPs are driven by shifting electrons. A well-known EEP is the piezoelectric polymer. EEPs can be applied in robotics. IEAs are actuated in a liquid electrolyte medium, which can be used in biological environments. The mechanism driving the actuation of IEAs is the movement of ions. Examples of IEAs include conductive polymers and polymer gels [3]. ER hydrogels are of interest in actuator applications due to their biocompatibility, water absorption properties, softness, and flexibility [4,5,6,7,8,9,10].

Gelatin is a bio-based elastomer selected as a soft actuation material. Gelatin is obtained by extraction from cow bones, pig bones, fish skins, and some insects [11,12,13,14]. Gelatin has a large number of polar functional groups that can be polarized under external electric field stimuli. However, the hydrogen bonds in gelatin limit the mobility of the polarized groups [15,16].

The distinctive capacity of graphene-based materials to facilitate smart material composites has been demonstrated. Two-dimensional graphene structures containing sp^2^-hybridized carbon atoms have demonstrated strong structural, chemical, and electrical capabilities. However, the dispersion of graphene in smart material composite limits the development of controllable actuation technology applications. Graphene oxide (GO) is one of the graphene derivatives. GO is highlighted as smart material filler because of the reactive functional groups on its surface. Synthetic chemistry could utilize the advantages of the structural properties of GO [17,18,19,20,21,22].

In this research work, we prepared GO as the additive particle in ER hydrogels. The effect of the KMnO_4_ ratio on the electrical properties of GO was investigated. The synthesized GO was used to fabricate a GO/GEL composite through a simple solvent-casting method. The ER properties of the GO/GEL composite with various amounts of GO were investigated under an external electric field. The GO/GEL composite had larger magnitude electromechanical responses as a movable entangled hydrogel composite than the GP/GEL composite and gelatin hydrogel. The expected outcome from this work is to fabricate a high actuation response composite with ease in a process that can be used for soft and high response actuations.

## 2. Materials and Methods

### 2.1. Materials

Graphite powder (AR grade, Sigma-Aldrich, St. Louis, MO, USA), sulfuric acid (AR grade, Vetec Quimica Fina company, Rio de Janeiro, Brazil), sodium nitrate (AR grade, Loba Chemie Pvt company, Mumbai, India), potassium permanganate, KMnO_4_ (AR grade, Ajax Finechem Pty company, Taren Point, NSW, Australia), hydrogen peroxide (AR grade, Fisher Scientific UK company, Loughborough, UK), and hydrochloric acid (AR grade, Loba Chemie Pvt company, Mumbai, India) were used to prepare the GO. Gelatin (AR grade, Acros Organics Chemical Products company, Geel, Antwerp, Belgium) was used as the ER hydrogel matrix. Poly(ethylene glycol) (AR grade, Sigma-Aldrich, St. Louis, MO, USA) was used as the plasticizer. Silicone oil (200 fluid 350 cSt, Ajax Finechem Pty company, Taren Point, NSW, Australia) was used as the liquid medium in the deflection experiment.

### 2.2. Synthesis of Graphene Oxide (GO)

The GO was prepared by Hummer’s method with modifications [23]. The effect of the potassium permanganate ratio on the electrical and physical properties of GO was investigated. The GP and KMnO_4_ were mixed at various GP/KMnO_4_ ratios (1:1, 1:2, 1:3, and 1:4 for GO1, GO2, GO3, and GO4, respectively). Graphite powder (1 g) was added to 23 mL of H_2_SO_4_. Then, the obtained solution was simultaneously mixed with 0.5 g of NaNO_3_ and KMnO_4_ (1 g, 2 g, 3 g, or 4 g) with temperature controlled below 20 °C. Afterwards, 46 mL of DI water was gradually added to the solution at 35 °C for 30 min, which was followed by 140 mL of DI water. Then, 10 mL of 30% *w/v* of hydrogen peroxide was dropped into the mixture and stirred at 35 °C for 2 h. The obtained GO was washed with 4 wt% hydrochloric acid solution and DI water until the washed water reached pH 7. Finally, the GO was freeze-dried for 29 h (G1700, Genvac). The success of the GO preparation was confirmed by FT-IR spectroscopy (PerkinElmer, model Spectrum Two, PerkinElmer, Inc., Waltham, MA, USA) and field emission scanning electron microscope (FE-SEM Zeiss AURIGA FE-SEM/FIB/EDX, A Carl Zeiss SMT AG Company, Oberkochen, Germany).

### 2.3. Preparation of Graphene Oxide/Gelatin (GO/GEL) Composite

To study the electroresponsive (ER) properties of the GO/GEL composite, gelatin was selected as the ER matrix because of its flexibility and highly hydrophilic nature [12,13,14]. The GO/GEL composite was prepared by mixing 4.5 g of gelatin into 45.5 mL of DI water at 80 °C for 45 min. Then, 0.225 g of ER particles (GO1, GO2, GO3, GO4, and GP) and 5 mL of poly(ethylene glycol) were added to the hydrogel solution at 25 °C for 1 h. Then, the GO/GEL mixing solution was poured into the mold (L 18 cm W 9 cm T 1 cm) and dried at room temperature for 96 h (GO1, GO2, GO3, GO4, and GP were 5% *w*/*w*).

The effect of the amount of GO on the ER properties of the GO/GEL composite was studied. The GO/GEL composite was prepared at various amounts of GO2 (1.25, 2.5, 5, 10, or 20% *w*/*w* were GO/GEL_2_1.25%, GO/GEL_2_2.5%, GO/GEL_2_5%, GO/GEL_2_5%, GO/GEL_2_10%, and GO/GEL_2_20%, respectively).

### 2.4. Electrical Properties of GO/GEL Composite

**Figure 1:**
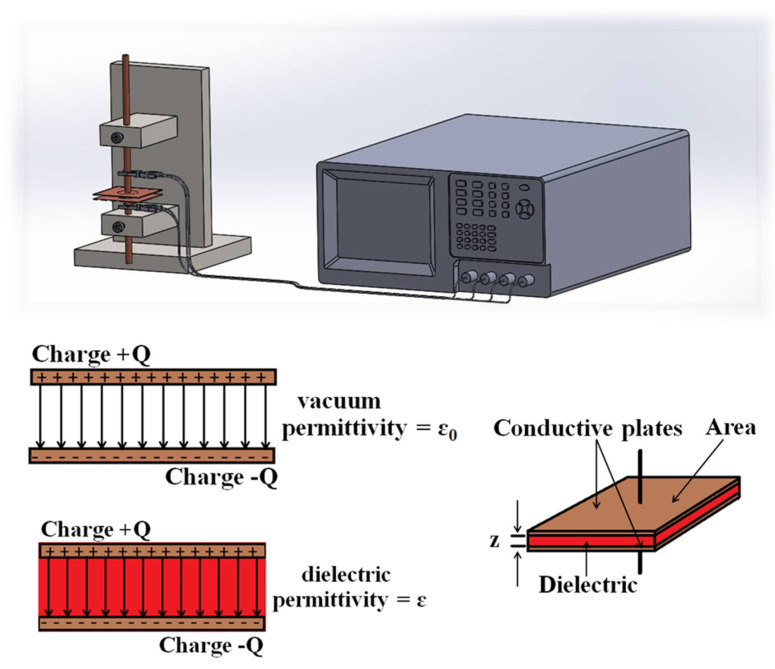
The schematic diagram of dielectric constant measurement.

The dielectric constant of the GO/GEL composite was measured by an LCR meter (LCR-8101G GW INSTEK, Good Will Instrument, Taipei, Taiwan). Resistance (R) and capacitance (C) were tested by placing a sample under an alternating current (AC) electric field, as demonstrated in Figure 1. The dielectric constant (ε) of the sample is equal to the ratio between the capacitance (C) and vacuum permittivity. The dielectric constant of the material was calculated from Equation (1).

(1) C=ε0ε
AZ

where ε_0_ is the permittivity of free space (8.854 × 10^−12^ c^2^/Nm); A is the area of copper electrodes; and Z is the distance between the plates [24].

### 2.5. The Deflection of GO/GEL Composite

**Figure 2:**
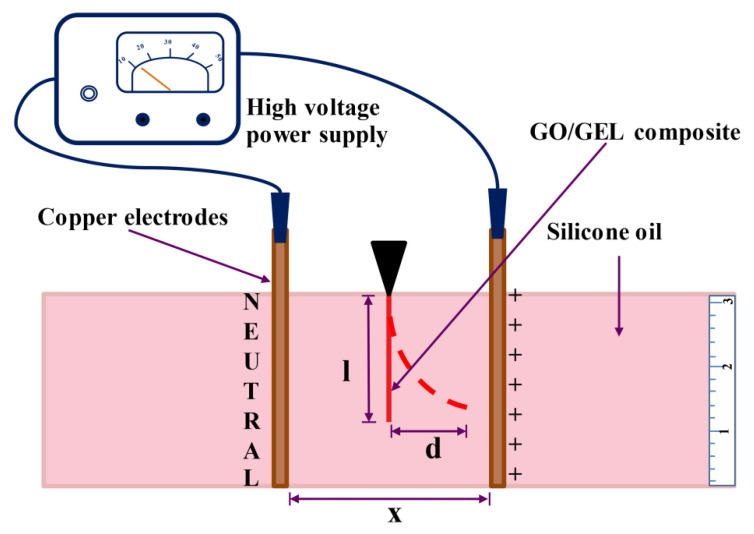
The deflection test of the GO/GEL composite.

The effects of the electrical conductivity and dielectric constant of the GO/GEL composite on ER properties were investigated in this research work. The deflection of the GO/GEL composite was characterized by providing an external electric field with strength ranging from 0 to 400 V/mm (HV 350 R 250 μA Power Supply High Voltage DC, Information Unlimited). The GO/GEL composite was clipped between the copper electrode plates dipped in silicone oil (viscosity of 350 cSt). The distances between the copper electrode plates were 4, 6, 8, and 10 cm. The schematic diagram of the deflection test is shown in Figure 2. The degree of deflection (θ) and responsive distance (d) of the GO/GEL composite were analyzed using Image J (version 1.52a, National Institutes of Health, Maryland, MD, USA). The dielectrophoresis force (F_d_) was calculated from Equation (2) [1,2,8,25,26,27,28,29].

F_d_ = F_e_ + mg(sin θ) − ρVg(sin θ) (2)

where F_e_ is the resisting elastic force (N), m is the mass of sample (kg), g is the gravity constant (9.8 m/s^2^), and ρ and V are the density and volume of displaced fluid silicone oil, respectively [1,2,8,25,26,27,28,29].

In the experiment, the elastic force (F_e_) was calculated from Equation (3).

(3) $$F_{e}=\frac{\mathrm{dEI}}{l^{3}}$$

where E is Young’s modulus; I is the moment of inertia; d is the distance of deflection; and l is the length of the specimen [1,2,8,25,26,27,28,29].

## 3. Results and Discussion

### 3.1. GO and GO/GEL Composite Characterization

**Figure 3:**
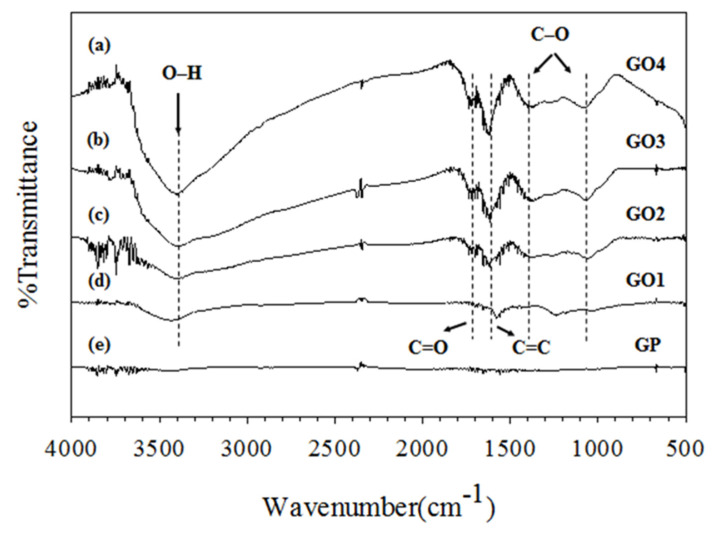
The FT-IR spectra of GO and GP.

To confirm the success of GO synthesis and study the effect of the GP/KMnO_4_ ratio on GO properties, the functional groups of GO (GO1, GO2, GO3, and GO4) were characterized. At different GP:KMnO_4_ ratios, different degrees of extensive oxidation occurred. The various functional groups of GO were observed. The successful GO preparation is shown in Figure 3. Synthesized GO showed characteristic peaks at 3050–3800, 1720, 1600–1650, 1410, and 1080 cm^−1^, corresponding to O−H stretching, carboxyl C=O stretching, aromatic C=C peak, O–H deformation, and C−O stretching, respectively. The results show that extensive oxidation increased when the KMnO_4_ ratio increased, which can be confirmed by the stronger and broader peaks. From the FT-IR spectra of GO, the GO structure showed various oxygen-containing functional groups such as carboxyl and hydroxyl groups. These results correspond to the previous work of Ickecan et al., Madhab Bera et al., Sudesh et al., and Zafer et al. [30,31,32,33,34,35].

**Figure 4:**
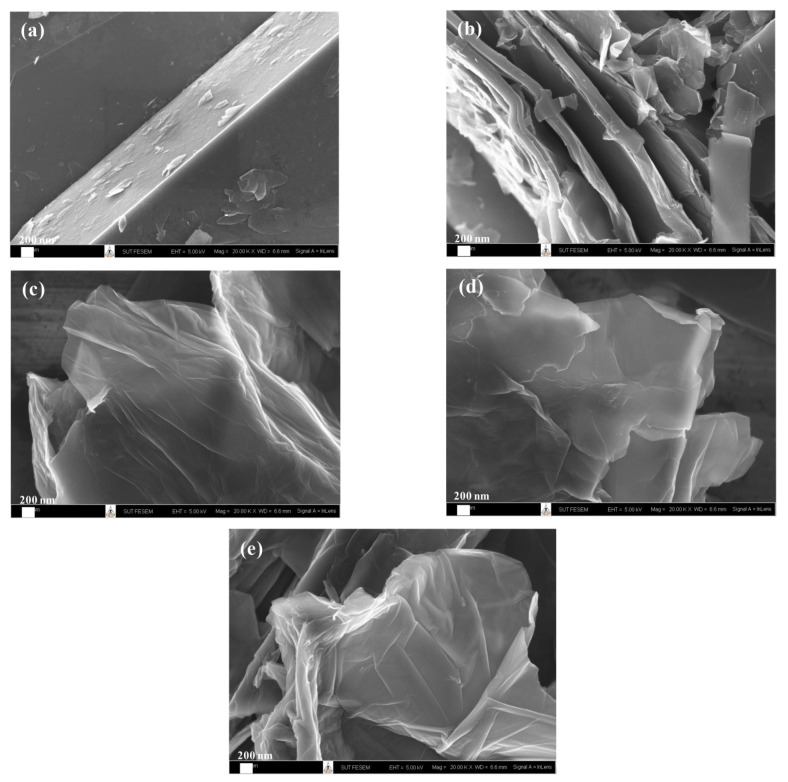
FESEM images of (**a**) GP, (**b**) GO1, (**c**) GO2, (**d**) GO3, and (**e**) GO4.

The morphology of synthesized GO at various GP/KMnO_4_ ratios (GO1, GO2, GO3, and GO4) was studied by field emission scanning electron microscope, as shown in Figure 4. Figure 4a showed the surface morphology of GP. The multi-stacked layers plate structure was observed, and the plate thickness is approximately 1 µm. The morphology of GO1, GO2, GO3, and GO4 is shown in Figure 4b–e, respectively. From FESEM images of GO1, the broke-up and exfoliated to thin plate was clearly observed. For GO2, GO3, and GO4, the randomly aggregated nanosheets was observed. The nanosheets thickness is approximately 1–40 nm. The separation breaks up, and exfoliated was increased with the GP/KMnO_4_ ratio due to the stronger oxidation reaction. When the degree of separation breaks up the GP increase, a few thin layers of GO were generated [31,36]. Paulchamy et al. [37] and Liu et al. [38] also reported that GO sheets were exfoliated. The exfoliated structure was micrometers in size.

To understand the ER properties, the electrical properties of the ER particles (GO and GP) and the gelatin hydrogel matrix (GEL) are the most important parameters. The electrical properties (capacitance, resistance, and dielectric constant) of GO, GP, gelatin powder (GE), GEL, and GO/GEL composite are tabulated in Table 1. The capacitance (C), resistance (R), and dielectric constant (ε) of the GO particle increased with the increasing ratio of GP/KMnO_4_ until it reached 1:2, after which they decreased. The amount of oxygen increased with the increasing GP/KMnO_4_ ratio, corresponding to an increased oxidation reaction. Thus, the concentration of O atoms in a C/O ratio increases with an increasing GP/KMnO_4_ ratio. The increase in C, R, and ε might be due to the exfoliation of the GO structure under the oxidation reaction. The exfoliation of GO decreases the electron mobility. At GP/KMnO_4_ ratios of 1:3 and 1:4, the intercalation of the hydroxyl group might increase, resulting in higher electron mobility. Thus, the GP/KMnO_4_ ratio of 1:2 is the most suitable ratio to improve the capacitance, resistance, and dielectric constant of GO as a non-conductive material in the ER composite [39,40].

The electrical properties of the GO/GEL composite, GP/GEL composite, and pristine GEL matrix were studied and are tabulated in Table 1. The GO/GEL and GP/GEL composites were prepared at 5% *w*/*w* of GO1, GO2, GO3, GO4, and GP for GO/GEL_1_5%, GO/GEL_2_5%, GO/GEL_3_5%, GO/GEL_4_5%, and GP/GEL_5%, respectively. The capacitance (C), resistance (R), and dielectric constant (ε) of the GO/GEL composite were lower than in the GO particles but higher than in gelatin and GP/GEL at the given number of particles. The results can be described by the mixture rule. In general, the composite electrical conductivity is predicted to be affected by GO composition [41].

To study the effect of the amount of ER particles on ER properties, GO2 was selected and mixed with GEL at various amounts of GO2 (1.25, 2.5, 5, 10, and 20% *w*/*w* for GO/GEL_2_1.25%, GO/GEL_2_2.5%, GO/GEL_2_5%, GO/GEL_2_10%, and GO/GEL_2_20%, respectively). The capacitance (C), resistance (R), and dielectric constant (ε) of GO/GEL_2_1.25%, GO/GEL_2_2.5%, GO/GEL_2_5%, GO/GEL_2_10%, and GO/GEL_2_20% are tabulated in Table 1. The C, R, and ε of the GO/GEL composite increase monotonically with the amount of GO. In general, the composite dielectric constant capacitance and resistance are likely affected by the GO composition, crystal structure, crystallinity, polarizability, phase transition, and microstructure morphology, which includes grain size, grain boundary, and pore size [41,42].

### 3.2. The Electroresponsive Properties of GO/GEL Composite

To study the ER properties of the GO/GEL composite, we studied the defection of the composite under an external electric field. The GO/GEL composites (0.5 width × 1.8 cm length) were dipped into silicone oil (viscosity of 350 cSt), and the distances between copper electrodes were 4, 6, 8, and 10 cm. The GO/GEL composites were actuated by an external electric field at 0–400 V/mm. The electrical stimuli cause an electroresponsive response by bending the composite toward positive electrodes. The degree of defection increases with the electric field strength (V/mm).

**Figure 5:**
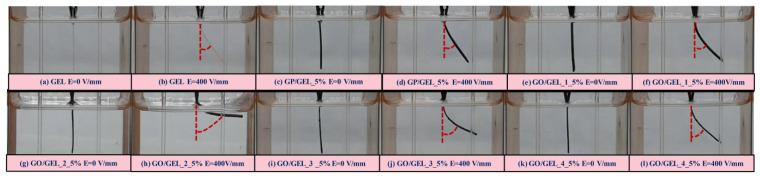
The degree of deflection for GO/GEL, GP/GEL, and GEL, with electric field strength of 0–400 V/mm and the electrode distance of 4 cm.

**Figure 6:**
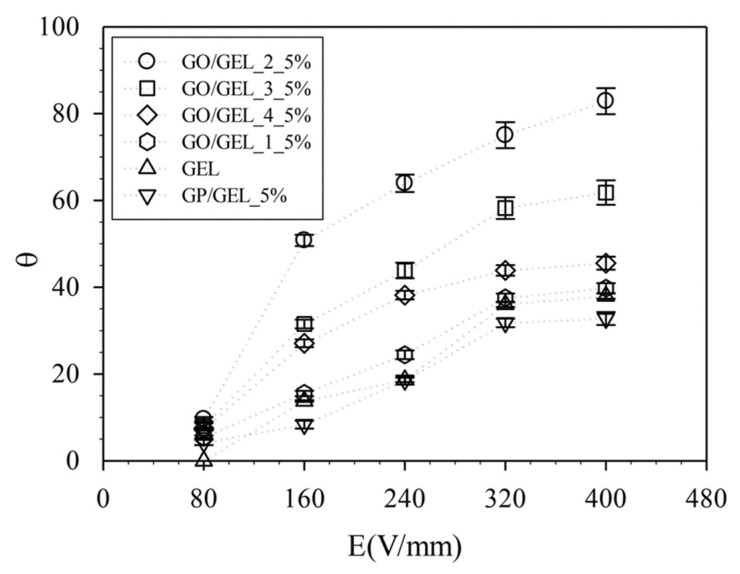
The electrical response of GO/GEL, GP/GEL, and GEL with electric field strength of 0–400 V/mm and the electrode distance of 4 cm.

To study the effect of particle electrical properties on deflection response, the deflection of GO (GO1, GO2, GO3, GO4,)/GEL, GP/GEL, and GEL under external electric field strength was characterized. The deflections of GO/GEL, GP/GEL, and GEL are shown in Figure 5 and Figure 6. Applying an external electric field strength of 400 V/mm and an electrode distance of 4 cm, the hydrogel’s free lower ends bent toward the positive electrode. The magnitude of deflection depended on the intensity of the electric field strength. The degrees of deflection of GO/GEL_1_5%, GO/GEL_2_5%, GO/GEL_3_5%, GO/GEL_4_5%, GP/GEL_5%, and GEL were 39.75°, 82.88°, 61.81°, 45.55°, 32.79°, and 37.87°, respectively. At the given amount of ER particles and electric field strength, the GO/GEL composite showed a higher degree of deflection than GP/GEL and GEL. GO/GEL_2_5% showed a higher degree of deflection than others. The deflection response mechanism can be described by the electrorepulsive force between the polarized carboxyl groups and ions of GO and electrodes, where the GO/GEL composite structures are negatively charged. Another mechanism of deflection is caused by the dielectrophoresis force generated by a polarizable body in an electric field. This occurs due to polarization; the polarized particles create attractive forces and form fibril structures [8,40,43,44,45].

**Figure 7:**
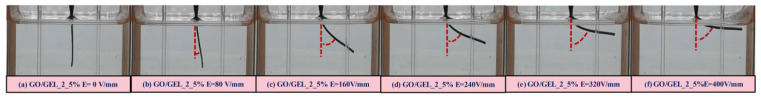
The degree of deflection for GO/GEL_2_5% with electric field strength of 0–400 V/mm and the electrode distance of 4 cm.

To investigate the effect of external electrical stimuli on electroresponsive properties, GO/GEL_2_5% was tested under electric strength of 0–400 V/mm. Figure 7 shows the degree of deflection of the GO/GEL composite under the external electric field. The degrees of deflection of GO/GEL_2_5% were (a) E = 0 V/mm (0°), (b) E = 80 V/mm (9.96°), (c) E = 160 V/mm (50.79°), (d) E = 240 V/mm (63.95°), (e) E = 320 V/mm (75.03°), and (f) E = 400 V/mm (82.88°). When the applied electric field strength increased, the deflection of the free end of the gel increased. A stronger dipole moment was produced under higher electric field intensity. Stronger dipole moments lead to a stronger attractive force between gels and electrodes, and thus, a higher degree of bending was observed [46].

**Figure 8:**
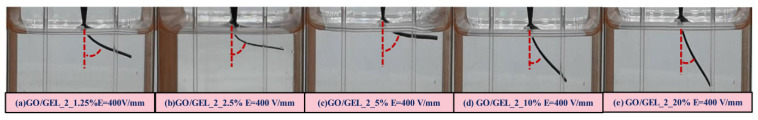
The degree of deflection for GO/GEL composites at various amounts of GO2 with electric field strength of 0–400 V/mm and the electrode distance of 4 cm.

**Figure 9:**
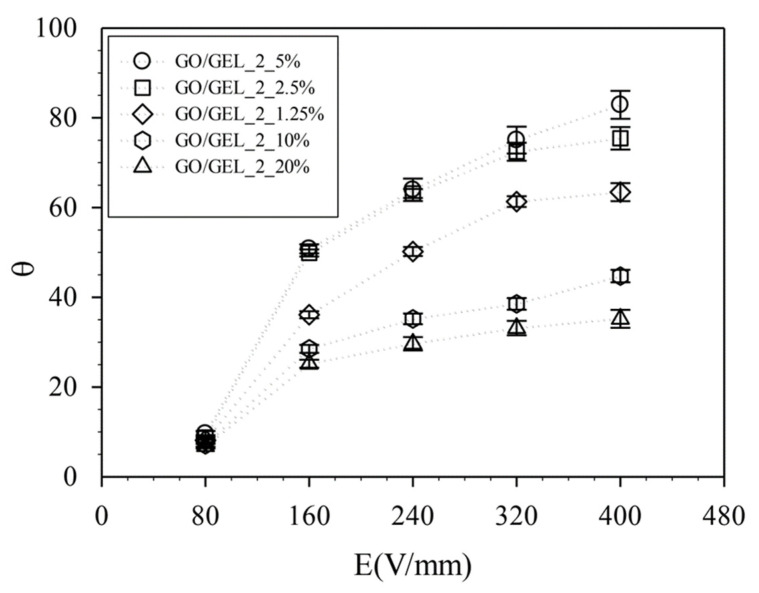
The electrical response of GO/GEL at various amounts of GO with electric field strength of 0–400 V/mm and the electrode distance of 4 cm.

To study the effect of the amount of GO particles on electroresponsive properties, we mixed GEL with various amounts of GO2 (1.25, 2.5, 5, 10, and 20% *w*/*w* for GO/GEL_2_1.25%, GO/GEL_2_2.5%, GO/GEL_2_5%, GO/GEL_2_10%, and GO/GEL_2_20%, respectively). The GO/GEL composites were characterized under an external electric field strength of 0–400 V/mm at a distance of 4 cm between the copper electrodes. Under the electric field strength of 400 V/mm, the degree of deflection was 63.44°, 75.43°, 82.88°, 44.73°, and 35.22° for GO/GEL_2_1.25%, GO/GEL_2_2.5%, GO/GEL_2_5%, GO/GEL_2_10%, and GO/GEL_2_20%, respectively, as shown in Figure 8 and Figure 9. The degree of deflection increased with GO content. Higher amounts of GO generated more polarized GO particles. A greater attraction force between the electrode and the polarized GO particles was obtained [41,44]. For neutral polymer gels, the bending actuation in a non-conducting medium depends on filler particles. In GO/GEL composites, the polarized GO particles cannot escape from the GEL hydrogel, all forces generating on the GO particles are immediately transferred to polymer molecular chains, resulting in motility or deformation [35,36,44]. Under higher electric field strength, the degree of deflection increases, as shown in Figure 9.

**Figure 10:**
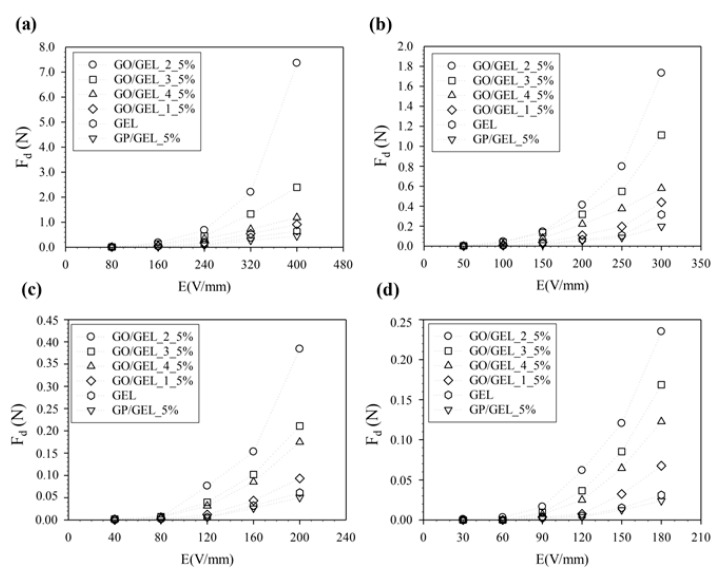
The F_d_ of GO/GEL, GP/GEL, and GEL under an external electric field of 0–400 V/mm at various electrode distances: (**a**) 4 cm, (**b**) 6 cm, (**c**) 8 cm, and (**d**) 10 cm.

The dielectrophoresis force (F_d_) that bends the GO/GEL composite was calculated by Equations (2) and (3), as tabulated in Table 2 and Figure 10. There is a strong attraction between the electrode and the polarized group, causing negative charges in the gelatin structure. When external electrical stimuli are applied, the gel’s free lower end deflects toward the cathode by a certain amount [26,35]. Concerning the effect of distance between various copper electrodes on the dielectrophoresis force (F_d_), F_d_ decreased with longer distances between various copper electrodes due to decreased electric field strength, which decreased the dielectrophoresis force.

**Figure 11:**
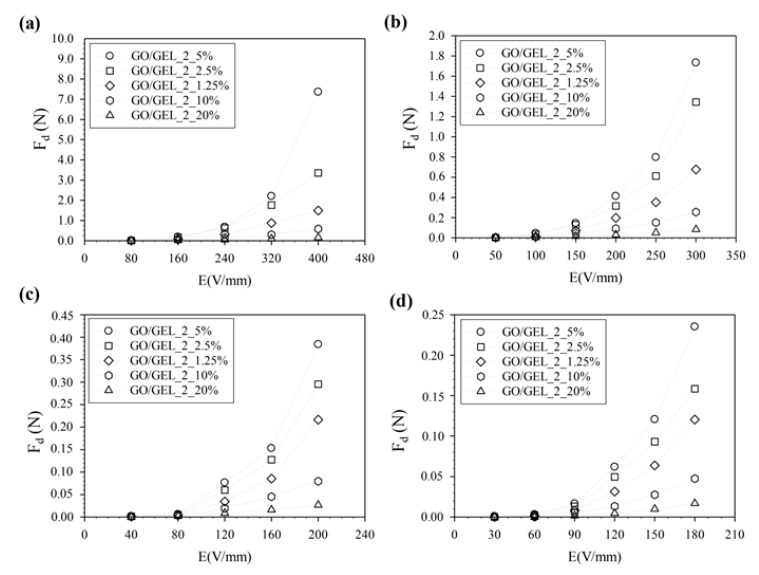
The F_d_ of the GO/GEL composite at various amounts of GO2 (1.25, 2.5, 5, 10, and 20% *w*/*w*) with an external electric field of 0–400 V/mm and various distances between copper electrodes: (**a**) 4 cm, (**b**) 6 cm, (**c**) 8 cm, and (**d**) 10 cm.

To study the effect of the amount of GO particles and distance between copper electrodes on F_d_ generation, the F_d_ of GO/GEL_2_1.25%, GO/GEL_2_2.5%, GO/GEL_2_5%, GO/GEL_2_10%, and GO/GEL_2_20% was calculated under an external electric field strength of 0–400 V/mm at various distances between copper electrodes of 4, 6, 8, and 10 cm, as tabulated in Table 3 and shown in Figure 11. The electroresponsive response and the calculated F_d_ increased with rising GO2 particle content up to 5% *w*/*w* and then decreased when the particle content was higher than 5% *w*/*w*. The distortion of the electron distribution and the charge displacement of ions inside the molecule caused the polarization of the carbonyl group. The highest F_d_ values were obtained at the proper GO content from polarized carboxyl groups.

An attractive force was generated between the polarized carbonyl groups in the gelatin molecule and the positive electrode. The net negative charges in the gelatin structure were generated from the carboxyl groups’ polarization. Thawatchai et al. studied the effect of temperature and electric field on the electromechanical properties of bio-compatible gelatins (Ala-Gly-Pro-Arg-Gly-Glu-4HypGly-Pro-). The F_d_ of gelatin was less than that of the polymer composites. The maximum deflection distance and F_d_ at an electric field strength of 600 V/mm were 1.28 mm and 4.859 μN, respectively [26]. Dai et al. studied the deflection response of poly(vinyl alcohol) (PVA) and poly(2-acrylamido-2-methyl-1-propanesulfonic acid) (PAMPS) under applied electrical stimuli. At an electric field strength of 400 V/mm, the deflection force of PVA/PAMPS was 4.9 mN [47]. Alici et al. [48] studied the actuation response of a polypyrrole/polyvinylidene fluorine composite. Under an electrical voltage of 1 V, the polypyrrole/polyvinylidene fluorine composite provided the output force of 0.6 mN.

## 4. Conclusions

In the present work, we studied the effect of the potassium permanganate ratio on electrical properties and the response to the electric potential difference by using gelatin as a hydrogel polymer. The capacitance, resistance, and dielectric constant had the same direction, increasing with the increase in the KMnO_4_ ratio to GO2 and then decreasing when the KMnO_4_ ratio was greater than 1:2. The GO/GEL composite (GO/GEL_2_5%) had the highest dielectric constant, capacitance, and resistance. The degree of bending and the dielectrophoresis force were investigated by studying the effects of external electrical stimuli to on bending the GO/GEL composite (GO/GEL_1_5%, GO/GEL_2_5%, GO/GEL_3_5%, and GO/GEL_4_5%) and the distance between various copper electrodes (4, 6, 8, and 10 cm) with E = 0–400 V/mm. The GO/GEL composite (GO/GEL_2_5%) showed the highest deflection angle (θ = 82.88°) and dielectrophoresis force (7.36 N). Regarding the effect of distance between various copper electrodes on the dielectrophoresis force (F_d_), the force decreased with longer distances due to lower electric field strength. The oxidation increased when the KMnO_4_ ratio increased, which was exemplified by strong and broad peaks. Thus, the result obtained from FT-IR affirmed the presence of various oxygen-containing functional groups such as carboxyl and hydroxyl within the GO structure. Thus, we demonstrate that our graphene oxide/gelatin composite candidate can be used as an electromechanical actuator in robot parts, artificial muscles, and MEM devices.

## Figures and Tables

**Table 1:** The dielectric constant of GO particles and matrices.

Sample	Particle			Matrix/Composite		
	C (pF)	R (MΩ)	ε_2_	C (pF)	R (MΩ)	ε_1_
Gelatin powder	8.65	44.95	5.98			
GEL				8.65	48.22	4.21
GP	95.88	N/A	8.19	-	-	-
GO1	101.24	11.94	10.25	-	-	-
GO2	132.02	30.98	13.67	-	-	-
GO3	126.04	28.57	12.84	-	-	-
GO4	114.97	24.22	11.18	-	-	-
GP/GEL_5%	-	-	-	34.93	21.77	4.67
GO/GEL_1_5%	-	-	-	47.26	32.83	6.88
GO/GEL_2_5%	-	-	-	71.98	41.74	9.17
GO/GEL_3_5%	-	-	-	68.80	41.64	8.69
GO/GEL_4_5%	-	-	-	66.47	39.15	8.11
GO/GEL_2_1.25%	-	-	-	42.69	40.87	6.85
GO/GEL_2_2.50%	-	-	-	47.32	41.24	7.47
GO/GEL_2_10%	-	-	-	76.15	42.83	10.48
GO/GEL_2_20%	-	-	-	84.70	43.63	11.38

**Table 2:** The Fd of GO/GEL, GP/GEL, and GEL under an external electric field of 0–400 V/mm.

Sample	Dielectrophoresis Force (F_d_)(N)/Electric Field Strength (E)			
	4 cm	6 cm	8 cm	10 cm
	400 V/mm	300 V/mm	200 V/mm	180 V/mm
GEL	0.65	0.32	0.06	0.03
GP/GEL	0.45	0.20	0.05	0.02
GO/GEL_1_5%	0.91	0.44	0.09	0.07
GO/GEL_2_5%	7.36	1.73	0.38	0.24
GO/GEL_3_5%	2.39	1.11	0.21	0.17
GO/GEL_4_5%	1.19	0.58	0.17	0.12

**Table 3:** The Fd of the GO/GEL composite at various amounts of GO2 (1.25, 2.5, 5, 10, and 20% *w*/*w*).

Sample	Dielectrophoresis Force (F_d_)(N)/Electric Field Strength (E)			
	4 cm	6 cm	8 cm	10 cm
	400 V/mm	300 V/mm	200 V/mm	180 V/mm
GO/GEL_2_1.25%	1.49	0.68	0.22	0.12
GO/GEL_2_2.5%	3.35	1.34	0.30	0.16
GO/GEL_2_5%	7.36	1.73	0.38	0.24
GO/GEL_2_10%	0.58	0.26	0.08	0.05
GO/GEL_2_20%	0.16	0.08	0.03	0.02

## Data Availability

Not applicable.
